# Recruitment, retention, and adherence of family caregivers: Lessons from a multisite trial

**DOI:** 10.17533/udea.iee.v41n2e04

**Published:** 2023-08-18

**Authors:** Leidy Johanna Rueda Díaz, Erika de Souza Guedes, Diná de Almeida Lopes Monteiro da Cruz

**Affiliations:** 1 Nurse, Doctor, Associate Profesor. Universidad Industrial de Santander, (Colombia). Email: ljruedad@uis.edu.co. https://orcid.org/0000-0001-5549-5926 Universidad Industrial de Santander Universidad Industrial de Santander Colombia ljruedad@uis.edu.co; 2 Nurse, Doctor, Universidade de São Paulo, (Brazil). Email: guedes_erika@hotmail.com https://orcid.org/0000-0001-5220-4142 Universidade de São Paulo Universidade de São Paulo Brazil guedes_erika@hotmail.com; 3 Nurse, Doctor. Senior Professor, Universidade de São Paulo (Brazil). Email: dinamcruz@usp.br https://orcid.org/0000-0003-1373-409X Universidade de São Paulo Universidade de São Paulo Brazil dinamcruz@usp.br

**Keywords:** caregivers, nursing, chronic disease, telephone, pragmatic clinical trial, cuidadores, enfermería, enfermedad crónica, teléfono, ensayo clínico pragmático, caregivers, enfermagem, doença crónica, telefone, cooperação e adesão ao tratamento

## Abstract

**Objective:**

To describe the recruitment, retention of family caregivers, and adherence to a telephone based intervention evaluated in a multi-site trial and provide recommendations for the design of future studies.

**Methods:**

A descriptive study based on a secondary analysis of a multi-site clinical development in Colombia and Brazil. Recruitment was measured by the number of participants eligible and consented. Retention was assessed by the percentage of participants with outcomes data at two follow-ups. The intervention adherence was measured by the percentage of the caregiver who received the intervention.

**Results:**

Of the family caregivers assessed, 63% were eligible, and 32.9% declined to be in the study for time restriction or no interest. In Colombia, the total retention rate of caregivers was 63.4% at the first follow-up and 48% at the second follow-up, while in Brazil was de 52.8% and 46.2%, respectively. At the end of the study, the sample comprised 28 and 70 caregivers in the intervention and control groups, respectively, for a retention rate of 47%. Of 104 family caregivers allocated to the intervention group, 42 (40.3%) received five sessions. Most reported not completing the Caregiver's Activity Diary.

**Conclusion:**

The recruitment of family caregivers, participant retention, and adherence to the telephone intervention was unsuccessful. Future studies should apply an assessment tool during the recruitment of family caregivers and replace the term "caregiver" with "care provider" in the material involved in the research; define a retention protocol before starting the study and involve family caregivers in the design of the interventions.

## Resumen

**Objetivo:**

Describir el reclutamiento, la retención y la adherencia de los cuidadores familiares en una intervención educativa telefónica evaluada en un ensayo multi-sitio y ofrecer recomendaciones para el diseño de futuros estudios.

**Métodos:**

Estudio descriptivo basado en un análisis secundario de un desarrollo clínico multicéntrico en Colombia y Brasil. El reclutamiento se midió por el número de participantes elegibles y que dieron su consentimiento. La retención se evaluó por el porcentaje de participantes con datos de resultados en dos seguimientos. La adherencia a la intervención se determinó por el porcentaje de cuidadores que recibieron la intervención.

**Resultados:**

De los cuidadores familiares evaluados, 63% fueron elegibles, y 32.9% declinaron participar en el estudio por restricción de tiempo o falta de interés. En Colombia, la tasa de retención total de cuidadores fue de 63.4% en el primer seguimiento y de 48% en el segundo, mientras que en Brasil fue de 52.8% y 46.2%, respectivamente. Al final del estudio, la muestra comprendía 28 y 70 cuidadores en los grupos de intervención y control, respectivamente, para una tasa de retención del 47%. De los 104 cuidadores familiares asignados al grupo de intervención, 42 (40,3%) recibieron cinco sesiones. La mayoría no completó el diario de actividades del cuidador.

**Conclusión:**

El reclutamiento de cuidadores familiares, la retención de participantes y la adherencia a la intervención telefónica no tuvieron éxito. Los estudios futuros deberían aplicar una herramienta de evaluación durante el reclutamiento de los cuidadores familiares y sustituir el término "cuidador" por "proveedor de cuidados" en el material empleado en la investigación; definir un protocolo de retención antes de iniciar el estudio e involucrar a los cuidadores familiares en el diseño de las intervenciones.

## Resumo

**Objetivo:**

Descrever o recrutamento, retenção e adesão de cuidadores familiares em uma intervenção telefônica avaliada num estudo clínico multi-site e oferecer recomendações para o desenho de estudos futuros.

**Métodos:**

Estudo descritivo baseado em análise secundária de um desenvolvimento clínico multicêntrico na Colômbia e no Brasil. O recrutamento foi medido pelo número de participantes elegíveis e que deram consentimento. A retenção foi avaliada pela porcentagem de participantes com dados de resultado em dois acompanhamentos. A adesão à intervenção foi determinada pela porcentagem de cuidadores que receberam a intervenção.

**Resultados:**

Dos cuidadores familiares avaliados, 63% eram elegíveis, e 32.9% se recusaram a participar do estudo por limitação de tempo ou falta de interesse. Na Colômbia, a taxa de retenção total dos cuidadores foi de 63.4% no primeiro acompanhamento e 48% no segundo, enquanto no Brasil foi de 52.8% e 46.2%, respectivamente. Ao final do estudo, a amostra foi composta por 28 e 70 cuidadores nos grupos intervenção e controle, respectivamente, para uma taxa de retenção de 47%. Dos 104 cuidadores familiares designados para o grupo de intervenção, 42 (40.3%) receberam cinco sessões. A maioria não preencheu o diário de atividades do cuidador.

**Conclusão:**

Recrutamento de cuidadores familiares, retenção de participantes e adesão à intervenção telefônica não tiveram sucesso. Estudos futuros devem aplicar uma ferramenta de avaliação durante o recrutamento de cuidadores familiares e substituir o termo 'cuidador' por 'fornecedor de cuidados' em material de pesquisa; definir um protocolo de retenção antes de iniciar o estudo e envolver os cuidadores familiares no desenho das intervenções.

## Introduction

Caring for a loved one can be physically and mentally quite taxing; hence, many family caregivers experience human responses that can affect their well-being and quality of life.^1^^,^^2^ One of family caregivers' most frequent nursing diagnoses is caregiver role strain. The prevalence of this diagnosis in caregivers varies from 73.8% to 98%.^3^ Family caregivers with role strain need practical and accessible interventions for coping with caregiving's physical and emotional aspects, like adapting to their role as care providers. In this sense, the telephone has been proposed as a resource for delivering interventions to family caregivers that could increase accessibility^4^^,^^5^ and affordability.^5^

We conducted a multi-site randomized clinical trial with two arms parallels (ReBEC, number UTN: U1111-1158-6171, RBR-8bvqz2) in Bucaramanga (Colombia) and São Paulo (Brazil) to evaluate the effectiveness of a psychoeducational intervention delivered by telephone to promote the adaptation of family caregivers of people with chronic disease with the nursing diagnosis caregiver role strain. The adaptation was considered to decrease the caregiver role strain and improve the well-being and quality of life. ^6^^,^^7^ The study period began in October 2014 and ended in November 2015. In both cities, there were difficulties in recruiting and retaining family caregivers and a lack of adherence to the intervention. These aspects are the object of analysis in the present paper. 

Recruitment refers to identifying or searching for potential participants who may be eligible for research and includes including participants in the study based on eligibility criteria.^8^ Retention is the maintenance of the participants included in the study until its completion,^8^ and adherence to the intervention is the which a participant follows the recommendations of a prescription or intervention.^9^These three elements are critical to validate the findings of any controlled clinical trial and yield evidence-based practice.^10^

Although the threats against participant recruitment and retention are significant when evaluating a remote intervention,^11^^,^^12^ studies that have testing interventions delivered exclusively by telephone for family caregivers do not detail the recruitment process^13^ or the strategies used for participant retention^13^^,^^14^ nor do they analyze adherence to the delivered intervention.^14^ Reporting these aspects is relevant so that the scientific community learns from the successes and errors of the studies, and consequently, future studies could be planned based on those lessons. Considering the above, this study aimed to describe the recruitment and retention of family caregivers and adherence to a telephone intervention evaluated in a multi-site trial and provide recommendations for the design of future studies.

## Methods

This study is descriptive, based on a secondary analysis of a multi-site clinical conducted in Bucaramanga (Colombia) and São Paulo (Brazil). The original study protocol was approved by the Committee of Ethics on Scientific Research of the Industrial University of Santander, code No. 7083; the committee of Ethics in Research of the School of Nursing of the University of São Paulo, code No.435.429; the committee of Ethics in Research of University Hospital-USP, Code No.547.201; and the committee of Ethics in Research the Hospital das Clínicas da Faculdade de Medicina-USP, code No. 776.413. All participants provided their signed informed consent forms before the study. 

### Recruitment

In the multisite clinical trial context, a sample size of 104 caregivers was calculated by country (52 for the control group and 52 for the intervention group) for a total of 208 family caregivers (104 for the control group and 104 for the intervention group). In Bucaramanga, caregivers were recruited in October and November 2014 in the Santander University Hospital (HUS) outpatient facility and the same institution's radiotherapy and chemotherapy unit. In São Paulo, caregivers were recruited between February and June 2015 in the outpatient facility of six healthcare institutions linked to the University of São Paulo. The inclusion criteria for participants were as follows: being a family caregiver of an adult with chronic disease with some degree of functional dependence, being 18 years or over, being able to read and write, providing care at home to the care recipient for more than one month, have telephone service and to present a minimum score of 14 points on the Caregiver Role Strain Scale. Exclusion criteria were the presence of speech or hearing limitations.

### Intervention

In the multi-site clinical trial context, family caregivers were randomly assigned to either control or experimental groups. The control group received the usual care, defined as the standard treatment provided by the health staff at the recruitment sites. The intervention group received the psychoeducational intervention "Taking care of me to take care of the other," consisting of five weekly telephone sessions. The intervention was developed using the Medical Research Council Framework. Topics covered in the sessions included: the meaning of being a caregiver, the deep breathing technique, the effects of care on health and well-being and the caregiver's rights, the feelings that the caregiver could experience due to caregiving, assertive communication, the problem-solving technique, caring for oneself (self-care) and time management. Moreover, each family caregiver received an activity diary containing the main content treated in each intervention session. In this diary, the caregiver should record the techniques taught by the nurses. Details regarded intervention are described in a publication.^7^ Eight Registered Nurses (3 Colombians and 5 Brazilians) delivered the intervention. None of them were responsible for usual care. All nurses had a baccalaureate degree and 1-15 years of experience caring for people with chronic diseases or family caregivers. Before implementing the intervention, the nurses received an intervention manual and 16-hour training from the principal investigator. The manual described the structure of each intervention session in detail, along with the nurses' instructions.^9^

### Implementation of a retention protocol

In the multi-site clinical trial context, a participant retention protocol was not considered a priori.

### Statistical analysis

Descriptive measures of sociodemographic characteristics of caregivers included are reported. Continuous variables are described through position statistics (mean, median) and dispersion (standard deviation and interquartile interval). Absolute and relative frequencies present the categorical variables. The characteristics of the caregivers were also compared between the groups using the Qui-square test for categorical variables, the Mann-Whitney test for the continuous variable Years of education, and the t-student test for the variable Age. Recruitment was measured by the percentage of participants eligible and consented. Retention was assessed by the percentage of participants with outcomes data at two follow-ups. Also, we compared demographic data between the family caregivers who remained in the study and those who were lost to follow-up. The intervention adherence was measured by the percentage of the caregivers who received 5, 4, 3, 2, or one intervention. We also calculated frequencies/percentages for describing the sessions received by family caregivers and completing the *Caregiver's Activity Diary* of the participants who received five intervention sessions. The means and standard deviations were calculated, and minimum and maximum values for the duration of calls and the number of days between sessions. All analyses were conducted using software R 3.2.2, and statistical significance was tested at level 0.05.

## Results

The demographic characteristics of participants are shown in Table 1. Family caregivers were mainly female, 178 (85.6%); daughters of the recipient care, 108 (51.9%); and homemakers, 93 (44.7%). Most caregivers, 152 (73.1%), lived with care recipients. The mean of the global support social index was 6.8 (19.5%). No relevant differences were found between the intervention and control groups at baseline for any sociodemographic, except employment status (*p*=0.01); therefore, homemakers were more frequents in the intervention group than in the control group.

**Table 1:** Sociodemographic characteristics of caregivers by study group. Bucaramanga, São Paulo 2014-2015

Variable	**Control Group (*n*=104)**	**Intervention Group (*n*=104)**	**Total (*n*=208)**
**Nationality; *n* (%)**			
Colombian	52 (50)	52 (50)	104 (50)
Brazilian	52 (50)	52 (50)	104 (50)
Gender			
Male	20 (19.2)	10 (9.6)	30 (14.4)
Female	84 (80.8)	94 (90.4)	178 (85.6)
Age (years); mean (SD)	47.8 (13.9)	47.5 (13.4)	47.6 (13.6)
**Relation with care recipient; *n* (%)**			
Sister-in-law	1 (1)	1 (1)	2 (1)
Grandson	2 (1.9)	2 (1.9)	4 (1.9)
Friend	2 (1.9)	5 (4.8)	7 (3.4)
Daughter-in-law	5 (4.8)	3 (2.9)	8 (3.8)
Nephew	4 (3.8)	4 (3.8)	8 (3.8)
Mother	7 (6.7)	4 (3.8)	11 (5.3)
Sister	9 (8.7)	5 (4.8)	14 (6.7)
Wife	20 (19.2)	26 (25)	46 (22.1)
Daughter	54 (51.9)	54 (51.9)	108 (51.9)
**Marital status; *n* (%)**			
Widowed	6 (5.8)	3 (2.9)	9 (4.3)
Divorced	10 (9.6)	8 (7.7)	18 (8.7)
Single	25 (24)	22 (21.2)	47 (22.6)
Married	63 (60.6)	71 (68.3)	134 (64.4)
Years of education; mean (SD)	11 [6 - 13]	11 [5 - 12]	11 [5 - 13]
**Employment status; *n* (%)**			
Retired	16 (15.4)	7 (6.7)	23 (11.1)
Unemployed	15 (14.4)	13 (12.5)	28 (13.5)
Freelancer	17 (16.3)	15 (14.4)	32 (15.4)
Employed	21 (20.2)	11 (10.6)	32 (15.4)
Homemarker	35 (33.7)	58 (55.8)	93 (44.7)
**Living with care recipient only; *n* (%)**	75 (72.1)	77 (74)	152 (73.1)
Index global support social; mean (SD)	65.7 (17.8)	61.9 (20.8)	63.8 (19.5)

### Recruitment of family caregivers

**Figure 1:**
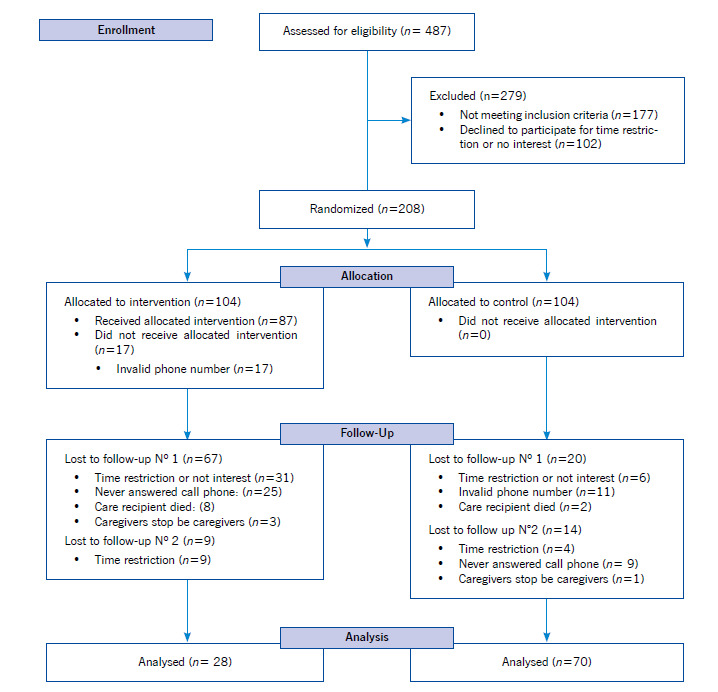
Schematic representation of the recruitment, allocation, and follow-up of caregivers

Of the 487 assessed, 310 family caregivers were eligible (63%), of whom 102 declined to be in the study for time restriction or no interest (32.9% of eligible). (Figure 1) shows a schematic representation of caregivers' recruitment, allocation, and follow-up.

### Retention of family caregivers

In Colombia, the total retention rate of caregivers was 63.4% (38% intervention group and 83% control group) at the first follow-up and 48% (29% intervention group and 67.3% control group) at the end of the second follow-up. The total retention rate of caregivers in Brazil was 52.8% (33% intervention group and 73% control group) at the first follow-up and 46.2% (25% intervention group and 67.3% control group) at the end of the second follow-up. At the end of the study, the sample comprised 28 and 70 caregivers in the intervention and control groups, respectively, for a retention rate of 47%. For both countries, there were statistically significant differences in losses to follow-up between the study groups, with more losses in the intervention group compared to the control group (*p*<0.001). However, there were no statistically significant differences between the number of losses to follow-up of caregivers of Brazilian nationality compared to those of Colombian nationality (*p*=0.87), neither in demographic data between family caregivers who remained in the studies contrasted with those who were lost to follow-up.

### Intervention adherence

Of 104 family caregivers assigned to the intervention group, 42 (40.3%) received five sessions, 14 (13.5%) received four sessions, 8 (8%) received three sessions, 8 (8%) received two sessions, 15 (14%) received one session, and 17 (16,2%) did not receive any intervention. When examining the interventions performed, the average duration of calls was 30 minutes (SD=14 min), with minimum and maximum values of 12 and 100 minutes, respectively. The mean number of days between two intervention sessions was 12 days (SD=11 days), with a minimum of 5 days and a maximum of 84 days between sessions.

Family caregivers were instructed to fill out the *Caregiver's Activity Diary* at the first telephone meeting. They should record the content learned during each intervention session and develop some activities on the topics covered in the sessions. The diary filling was evaluated at the beginning of the second, third, fourth, and fifth intervention sessions. As observed in Table 2, most caregivers who filled the five intervention sessions reported not having completed the *Caregiver's Activity Diary* prior to the intervention session.

**Table 2:** Frequency of filling of Caregiver's Activity Diary of 42 participants who received five intervention sessions

Frequency	Session 1 *n* (%)	Session 2 *n* (%)	Session 3 *n* (%)	Session 4 *n* (%)
Diary filling of the family caregiver				
Never	27 (64)	22 (52)	26 (62)	30 (71)
Rarely	10 (24)	10 (24)	4 (10)	5 (12)
Sometimes	3 (7)	7 (17)	10 (24)	4 (10)
Very often	2 (5)	2 (5)	2 (5)	2 (5)
Always	0 (2)	1 (2)	0 (0)	1 (2)
Practice without diary filling	6 (22)	7 (32)	5 (1)	3 (10)

## Discussion

The findings of this study show the challenges faced in a multisite clinical trial regarding the recruitment and retention of family caregivers in the research and their adherence to the evaluated intervention.

### Recruitment family caregivers

Although the sample size was reasonable, recruiting caregivers was difficult, especially in the study developed in São Paulo. Among the situations that limited recruitment, it is highlighted that many caregivers did not recognize themselves as caregivers despite reporting offering care to their family members. Many who expressed themselves as family caregivers did not accept participating in the research due to a lack of interest or time. Time limitations have been the leading cause of refusal and dropout reported by other studies involving family caregivers.^15^^-^^18^ Family caregivers, day by day, must face the demands of caring for their loved ones and others' responsibilities, which cause feelings of overload^19^^,^^20^ and lack of time,^21^ making them restrict participation in research. 

One study^22^ investigated the factors related to the decision of family caregivers to participate in a relaxation therapy intervention. The authors reported that caregivers who agreed to participate in the research were those who, despite feeling overwhelmed, recognized or admitted their own need to be helped or perceived that the research could benefit them by helping to improve their skills as caregivers or perceived that with their participation they would be contributing to the research on caregivers.^22^ In this sense, it is possible that caregivers potentially eligible for the research did not accept to participate because, despite the tension of the role, they did not perceive the need to receive care from nursing professionals or did not perceive benefits resulting from participation in the study. The recruitment of family caregivers in this research showed that they are a population of difficult access.

Other authors state that its recruitment for intervention studies is challenging.^23^^,^^24^ Therefore, researchers who intend to develop research involving family caregivers must implement strategies that enable their identification and engagement. To facilitate their identification, we suggest applying an assessment tool. Another suggestion to circumvent the lack of self-recognition as a caregiver would be to replace the term "caregiver" with "care provider" in the material involved in the research. This strategy proved effective when implemented in a clinical trial with family caregivers.^25^ In the case of engagement, to facilitate it, the team responsible for approaching potential participants must highlight and reinforce the gains that the family caregiver, the care receiver, and other caregivers can obtain from their participation in the research.

### Retention of family caregivers

Caregiver in the multi-site clinical trial was low, and in part, it can be explained by the lack of an a priori retention protocol. Hence the relevance of defining, before starting the execution of the research, strategies that avoid the interruption of the participation of family caregivers in the studies and, consequently, the abandonment and loss of follow-up. An interesting aspect to highlight is that despite the caregivers being aware of their right to withdraw from participating in the research, many chose not to answer the calls again, despite agreeing to a telephone meeting. Those who expressed their desire to give up argued did not have time to take the calls. 

There is also the possibility that attributes of the therapeutic relationship established between nurses and caregivers have not favored the retention of caregivers in the intervention program. A previous study reported that the caregivers' relationship with the research staff influenced their retention in a large relaxation therapy intervention study.^22^ Caregivers who felt respected, cared for and appreciated by the research staff completed the intervention. ^22^ The evaluation of the intervention's fidelity made it possible to identify that there was variation in the nurses' skills of empathy, sensitivity, the transmission of trust, and credibility, as well as to identify that the specific contents of the Intervention Program Caring for Me to Caring for the Other were offered to most caregivers.^26^ It is possible that the nurses' training was insufficient to prepare them for the role of interventionists within the research, thus affecting the nurse-caregiver relationship and, consequently, the retention of caregivers in the intervention program. Hence, the intervention's fidelity should be monitored throughout the study's development and interventionists' training whenever necessary.

### Adherence to intervention

During the development of the intervention sessions, it was noticed by the intervening nurses that most caregivers had a little proactive and disinterested attitude. It also evidenced their difficulty in "disconnecting" from their surroundings while answering calls, which generated frequent interruptions during the sessions. Low adherence to the intervention program raises questions about its feasibility and acceptance. Most caregivers do not use the theorized techniques to promote adaptation, such as deep breathing, progressive relaxation, and problem-solving techniques. This may indicate the need for more significant reinforcement for the practice of these techniques than was performed in this study. The participants' adherence to the intervention may be due to caregivers not recognizing the potential benefits of recommended practices and, therefore, not performing them or that the recommendations are not feasible. Unfortunately, the satisfaction of family caregivers with the intervention program or the perception of its usefulness was not evaluated. Data of this type could better inform the interpretation of adherence and clinical trial results.

A limitation of the intervention was the difficulty of agreeing on a time convenient for the caregiver and the nurse to carry out the session. Many caregivers expressed having time for intervention sessions late at night; in contrast, most nurses who delivered the intervention were available during the daytime. This same limitation was reported in another telephone intervention study.^14^ To overcome this difficulty, professionals responsible for delivering the intervention must be available 24 hours a day, seven days a week.

Although the telephone intuitively seems convenient for family caregivers to participate in psychoeducational interventions, it is necessary to investigate whether this medium is adequate for family caregivers in developing countries. It is also necessary to consider the preferences and interests of family caregivers regarding the content and duration of interventions. In this sense, we call on researchers to involve family caregivers in designing interventions and be concerned with obtaining evidence of feasibility, acceptability, and meaning before testing effectiveness. In order to evaluate the effectiveness, the intervention manual must be detailed and provide a script for the intervention application, as in this research. The intervention's fidelity must be measured to identify which elements of the intervention were effectively offered and, in this way, allow more excellent reliability of the results. 

For future research, it is necessary to include the care recipient in the intervention development whenever his health status and cognition allow. The finding of a meta-analysis of psychoeducational interventions for people with chronic diseases and their family caregivers showed that couples' interventions positively improved the care recipient's health and decreased the family caregiver's burden.^27^

## Conclusion

In the multisite clinical trial context, the recruitment of family caregivers, participant retention, and adherence to the telephone intervention was unsuccessful. In this sense, we highlight that the caregiver's non-recognition of themselves as family caregivers, did not respond to phone calls, had difficulties agreeing on a convenient time for the nurse to carry out the session, and did not use techniques such as deep breathing, progressive relaxation, and problem-solving. To mitigate these difficulties, we recommend applying an assessment tool during the recruitment of family caregivers and replacing the term "caregiver" with "care provider" in the material involved in the research; define a retention protocol before starting the study and involve family caregivers in the design of the interventions and worry about obtaining evidence of feasibility, acceptability, and significance before testing effectiveness.
